# iTRAQ Identification of Candidate Serum Biomarkers Associated with Metastatic Progression of Human Prostate Cancer

**DOI:** 10.1371/journal.pone.0030885

**Published:** 2012-02-15

**Authors:** Ishtiaq Rehman, Caroline A. Evans, Adam Glen, Simon S. Cross, Colby L. Eaton, Jenny Down, Giancarlo Pesce, Joshua T. Phillips, Ow Saw Yen, George N. Thalmann, Phillip C. Wright, Freddie C. Hamdy

**Affiliations:** 1 Department of Human Metabolism, The Medical School, The Mellanby Centre for Bone Research, University of Sheffield, Sheffield, United Kingdom; 2 Biological and Environmental Systems Group, Department of Chemical and Biological Engineering, ChELSI Institute, University of Sheffield, Sheffield, United Kingdom; 3 Academic Unit of Pathology, Department of Neuroscience, Faculty of Medicine, Dentistry and Health, University of Sheffield, Sheffield, United Kingdom; 4 Department of Urology, City Hospital, Birmingham, United Kingdom; 5 Department of Urology, Anna-Seiler-Haus, University of Bern, InselspitalInselspital, Bern, Switzerland; 6 Nuffield Department of Surgical Sciences, University of Oxford, John Radcliffe Hospital, Oxford, United Kingdom; University of Central Florida, United States of America

## Abstract

A major challenge in the management of patients with prostate cancer is identifying those individuals at risk of developing metastatic disease, as in most cases the disease will remain indolent. We analyzed pooled serum samples from 4 groups of patients (n = 5 samples/group), collected prospectively and actively monitored for a minimum of 5 yrs. Patients groups were (i) histological diagnosis of benign prostatic hyperplasia with no evidence of cancer ‘BPH’, (ii) localised cancer with no evidence of progression, ‘non-progressing’ (iii) localised cancer with evidence of biochemical progression, ‘progressing’, and (iv) bone metastasis at presentation ‘metastatic’. Pooled samples were immuno-depleted of the 14 most highly abundant proteins and analysed using a 4-plex iTRAQ approach. Overall 122 proteins were identified and relatively quantified. Comparisons of progressing *versus* non-progressing groups identified the significant differential expression of 25 proteins (p<0.001). Comparisons of metastatic *versus* progressing groups identified the significant differential expression of 23 proteins. Mapping the differentially expressed proteins onto the prostate cancer progression pathway revealed the dysregulated expression of individual proteins, pairs of proteins and ‘panels’ of proteins to be associated with particular stages of disease development and progression. The median immunostaining intensity of eukaryotic translation elongation factor 1 alpha 1 (eEF1A1), one of the candidates identified, was significantly higher in osteoblasts in close proximity to metastatic tumour cells compared with osteoblasts in control bone (p = 0.0353, Mann Whitney U). Our proteomic approach has identified leads for potentially useful serum biomarkers associated with the metastatic progression of prostate cancer. The panels identified, including eEF1A1 warrant further investigation and validation.

## Introduction

In Europe and the US, prostate cancer is the second most common cancer diagnosis and the third most common cause of cancer-related deaths in men [1], [2]. Moreover, the incidence has increased since the widespread introduction of prostate specific antigen (PSA) testing [3]. Most patients with prostate cancer are diagnosed at an early stage, but even with screening over 7% of cases develop metastatic disease [4]. In men with distant metastasis the prognosis is poor, with an average survival of 24 to 48 months. Bone is the most common site for prostate cancer metastasis and is associated with bone pain, spinal cord compression and marrow failure [4]. Currently, bone metastatic lesions are determined by imaging such as isotope bone scanning, however, the identification of a serum based biomarker(s) for predicting the susceptibility of patients to develop bone metastasis could enable a more accurate clinical assessment of the disease and help guide therapy.

The diagnosis of prostate cancer is most commonly made by a triad of serum prostate specific antigen (PSA) measurements, digital rectal examination (DRE), and histological assessment of transrectal ultrasound (TRUS) guided biopsy material [5]. Although PSA is a FDA approved biomarker for prostate cancer detection, its usefulness is controversial as it has been shown to be unreliable for disease diagnosis, and in particular for distinguishing indolent from aggressive forms of the disease [6], [7]. Additionally, PSA is associated with a high degree of false-positive and false-negative test results, as levels may be elevated in non-cancer conditions of the prostate, including benign prostatic hyperplasia (BPH). Thus, additional biomarkers are urgently needed which could improve the diagnostic specificity of PSA and predict the likelihood of disease progression.

Blood and its products, such as plasma and serum are ideal fluids for the identification of cancer biomarkers since they contain proteins both secreted and shed from cancer cells, combined with the ease of sampling. However, the variable composition and large dynamic range of proteins present in plasma (estimated to be 10^10^ orders of magnitude or more), pose formidable challenges in identifying clinically relevant biomarkers amongst the background of abundant proteins such as albumin, immunoglobulin and transferrin [8]. Of the 22 or so most abundant proteins in plasma, these constitute more than 99% of the mass of the total plasma proteins, while the remaining 1% are thought to be composed of the medium/low abundance proteins and include the biomarker pool [9]. The large orders of magnitude in protein concentration have hampered previous mass spectrometry based efforts aimed at identifying clinically relevant biomarkers, mainly due to a suppression of ionization of the low abundance proteins by the higher abundance proteins [10]. However, prior removal of some of the most highly abundant proteins has been shown to improve the detection of relatively lower abundant proteins [11], [12]. Although there are many different protein fractionation methodologies based on differences in molecular weight, shape, charge, pI, hydrophobicity and affinity through specific biomolecular interactions, it has been reported that high abundance protein separation using the antibody based IgY-12 immunodepletion system is highly reproducible [13].

Amongst the proteomic technologies used for biomarker identification, ‘isobaric Tags for Relative and Absolute Quantitation’ (iTRAQ) has the advantages of being relatively high throughput, and can simultaneously provide information on peptide quantitation and identification, as previously reported by us and others [14]–[16]. Briefly, in a typical workflow samples are reduced, alkylated and proteolytically digested to generate peptides. The peptides are labeled with a set of iTRAQ reagents (in a 4 or 8-plex format), pooled and fractionated by strong cation exchange (SCX). The fractions are then analyzed by liquid chromatography tandem mass spectrometry (LC-MS/MS), with the resultant mass spectra providing sequence information (from the peptide fragments), and relative quantification (from the reporter group ions).

In an effort to identify novel proteins associated with the metastatic progression of human prostate cancer, we have performed a 4-plex iTRAQ analysis using pooled serum samples collected prospectively from 4 well defined groups of patients who were actively monitored for at least 5 years, and selected to represent the spectrum of prostatic disease. Following data analysis, a number of candidates were found to be significantly differentially expressed in cancer samples compared with benign samples. One of the candidates identified as being significantly up-regulated in cancer groups was eukaryotic translation elongation factor 1 alpha 1 (eEF1A1), and was further investigated by immunohistochemistry using prostate tissue samples from localized and metastatic cases. The biomarker leads identified in our ‘discovery’ phase study, including eEF1A1 are discussed in relation to their significance to prostate cancer progression.

## Materials and Methods

### Patients and serum collection

Peripheral blood was collected prospectively from patients attending the Urology clinic at the Royal Hallamshire Hospital at the time of their initial visit, following written informed consent. Blood sample collection was approved by the Ethics Committee of the University of Sheffield. Serum was collected by allowing the blood to coagulate for 30 min, centrifuged at 1,200× g for 10 min at 4°C and then stored at −80°C in 100 µl aliquots. All blood samples were collected prior to the administration of any treatment. Twenty serum samples were carefully selected to represent the various stages and grades of prostate disease and pooled (n = 5 patients/group), to form 4 patient groups. All patients were actively monitored for at least 5 years from the time of their initial blood sampling. The 4 patient groups were: Group 1: histological diagnosis of benign prostatic hyperplasia ‘BPH’, with no evidence of cancer by at least 2 independent sets of prostatic biopsies, and a PSA level below 10 ng/ml (mean age of 61 yrs). Group 2: histological diagnosis of prostate cancer with a PSA level below 10 ng/ml, and no evidence of a rising PSA following 5 yrs active monitoring - ‘non-progressing’ group, (mean age of 67 yrs); Group 3: histological diagnosis of clinically localised cancer with an initial PSA level below 13 ng/ml, followed by 3 consecutive rises in PSA levels during 5 yrs of active monitoring -‘progressing’ group, (mean age of 69 yrs); Group 4: patients with a PSA>19 ng/ml and evidence of bone metastasis from a positive radionucleotide bone scan - ‘metastatic’ group, (mean age of 73 yrs). The differences in the median ages of the patients were found not to be statistically significant between the 4 groups (p = 0.146, Kruskal-Wallis test). The disease characteristics of the 20 patients comprising the 4 groups are shown in Table S1.

### Patient tissue material

Tissue microarrays (TMAs), comprised of 56 cases of prostatic adenocarcinoma ranging in single Gleason grades (i.e. grade 2, n = 5; grade 3, n = 32; grade 4, n = 9; grade 5, n = 10), and 40 cases of adjacent non-malignant tissue, and were constructed as previously described [17], [18]. An additional 23 cases of bone biopsies from patients both with and without prostate cancer skeletal metastasis were obtained by 8 mm trephine biopsy performed under general anesthesia. Informed patient consent and Ethics Committee approval was obtained prior to the study (South Sheffield Research Ethics Committee, SSREC/02/155 and 00/172).

### Cell lines

Human prostate cancer cell lines LNCaP, PC-3, DU145 and VCaP were purchased from the American Type Culture Collection (ATCC), (http://www.lgcstandards-atcc.org/). DuCaP cells were obtained via the PRIMA project consortium (http://www.primaproject.org/participants.php). The LNCaP-Pro5, LNCaP-LN3, PC-3M and PC-3M-LN4 cells were a kind gift from Dr. Curtis Pettaway (University of Texas, M.D. Anderson Cancer Centre), [19]. The LNCaP-C4-2 and LNCaP-C4-2B cell lines were obtained from Prof. George Thalmann [20]. The TE-85 osteosarcoma cells were a kind gift from Prof. J.A. Gallagher, University of Liverpool. All prostate cancer cell lines were cultured as previously described and confirmed to be free from Mycoplasma [15].

### IgY-14 affinity depletion of serum samples

Pooled serum samples were depleted of the 14 most common plasma proteins using the Seppro IgY-14 depletion system [21]. Previous studies have shown that serum pooling followed by depletion of the most highly abundant proteins is an effective strategy to reduce the dynamic range of proteins, and thus enhance the identification of serum biomarkers, as demonstrated using the quantitative proteomic method of iTRAQ(R) [22]. Serum samples from 5 patients representing each of the 4 patient groups were pooled in equal volumes to give a total volume of 200 µl for each group (40 µl of each sample). The pooled serum samples were shipped on dry ice to Genway (Digilabs Biovision, Germany), for immuno-depletion using the Seppro Ig-Y14 system. The flow-through fraction (depleted of albumin, immunoglobulin IgG, fibrinogen, transferrin, IgA, IgM, haptoglobin, alpha2-macroglobin, alpha1-acid glycoprotein, alpha1-antitrypsin, Apo A-I HDL, Apo A-II HDL, complement C3 and LDL (ApoB)), was used for subsequent iTRAQ analysis.

### iTRAQ sample labelling and SCX fractionation

Prior to iTRAQ analysis, samples were concentrated and buffer exchanged using 5 kDa molecular weight cut-off spin concentrators (Millipore). The samples were buffer exchanged three times against 500 µl of 1 M triethylammoniumbicarbonate (TEAB) buffer and concentrated to a volume of approximately 80 µl. Samples were labelled with the iTRAQ reagents according to the manufacturers instructions, and as previously described [14], [15]. Each sample was labelled with one of the four iTRAQ reagents (iTRAQ reporter ions of 114.1, 115.1, 116.1, 117.1 mass/charge ratio). The tag labelling order was BPH- 117; localised non-progressing cancer-116; progressing cancer-115; metastatic disease-114). Labelled samples were pooled and fractionated by strong cation exchange (SCX), using a BioLC HPLC column (Dionex, Surrey, UK), and analyzed by LC-MS/MS as previously described [14], [15].

### Tandem mass spectrometry analysis

Mass spectrometry (MS) was performed using a QStar XL Hybrid ESI Quadrupole time-of-flight tandem mass spectrometer, ESI-qQ-TOF-MS/MS (Applied Biosystems, Framingham, MA; MDS-Sciex, Concord, Ontario, Canada), coupled with an online capillary liquid chromatography system (Famos, Switchos and Ultimate from Dionex/LC Packings, Amsterdam, The Netherlands). The dried samples were resuspended in 60 l of 3% acetonitrile and 0.1% formic acid ready for the MS, and 10–15 l (depending on the peptide concentration as seen in the peak intensity of the SCX chromatogram) were injected to the nano-LC-ESI-MS/MS system for each analysis. Initial separation took place on a PepMap C_18_ RP capillary column (LC Packings) with a constant flow rate of 0.3 l/min. LC buffers A and B were made up as 3% acetonitrile, 0.1% formic acid and 97% acetonitrile, 0.1% formic acid, respectively. The gradient was started as 97% buffer A and 3% buffer B for 3 minutes, followed by 3 to 25% buffer B for 120 minutes, 90% buffer B for 7 minutes and finally 97% buffer A for 7 minutes. Data acquisition in the mass spectrometer was set to the positive ion mode, with a selected mass range of 350–1800 m/z. Tandem mass spectrometry was performed on peptides with +2, +3, +4 charge states across a scan range of 65–2000 m/z.

### Protein identification and relative quantification

Protein identification and relative quantification was carried out as previously described [23], [24]. Identification of peptide precursor and fragments was performed by database searching against the Swiss-Prot and Trembl *Homo sapiens* protein database (41070, 71449 ORFs respectively, downloaded from UniProt, May 2010). Parameters for searching were set up as follows: MS tolerance was 0.4 and MS/MS tolerance were set at: peptide tolerance 0.4 Da, charge +2, +3 and +4, min peptide length, z-score, max p-value and AC score were 6, 6, 10^−6^ and 6 respectively. Phenyx default ‘turbo’ scoring was enabled with mass tolerance restriction of 0.1 Da for MS and MS/MS and the minimum percentage of the peptide sequence coverage by b^+^ (b), b^2+^ (b++), y^+^ (y) or y^2+^ (y++) fragment series was set to the default value of 20%. Target database search space was restricted to tryptic peptides with a maximal of 1 miscleavage. Modifications were set as: 4-plex iTRAQ mass shifts (+144 Da, K and N-term), methylthiol (+46 Da) and oxidation of methionine (+16 Da). False discovery rates (FDR) were estimated using a concatenated target-decoy database as described by Elias and Gygi [25]. Protein changes were qualified using a t- test algorithm developed in house [23].

### Immunohistochemistry for eEF1A1

Immunohistochemistry was performed essentially as previously described [18]. Sections of bone and prostatic tissues were cut (4 m) and mounted on superfrost slides (VWR International, Germany). Slides were incubated with mouse monoclonal anti-eEF1A1 antibody (Santa-Cruz Biotechnology, CA, USA Cat. N^o^ sc21758), at 0.4 µg/ml in 2% horse serum overnight at 4°C. Sections were washed twice in PBS-Tween 20 (PBST), and incubated for 30 min with anti-mouse IgG ImmPRESS HRP (Vector Laboratories, Cat. N^o^ MP-7402). After further washing in PBST, localisation of antibody/antigen complex was visualized using the ImmPACT DAB system (Vector Laboratories, Cat. N^o^ SK-4105). Control sections were incubated with anti-mouse IgG isotype control (Vector laboratories I-2000), diluted to 0.4 µg/ml in 2% normal horse serum. eEF1A1 immunostaining was assessed for both intensity and cellular localization by an experienced histopathologist (Dr. Simon Cross), who was blinded to the study. Each case was assigned a staining intensity, ranging from 0–3, where 0 = absent; 1 = weak; 2 = moderate and 3 = Intense staining as previously described [18].

### Western blotting

Western blotting was performed essentially as previously described [26]. Anti-EF-Tu goat polyclonal IgG primary antibody was used at a concentration of 1∶1000 (Santa Cruz, Cat. N^o^. sc12990). Secondary antibody was HRP-conjugated rabbit anti-goat (Abcam), and was used at a concentration of 1∶1400 (Abcam). Dual color precision plus molecular weight markers were used for size estimation (Bio-Rad, Hertfordshire, UK).

### Reverse-Transcription PCR and Sequencing

Total RNA was extracted from prostate cancer cell lines using TRI reagent (Sigma-Aldrich, UK), according to the manufacturer's instructions. The RNA was quantified spectrophotometrically and 2 µg was reverse transcribed into cDNA using the SuperScript III Reverse Transcriptase kit with 250 ng of random primers, according to the manufacturer's instructions (Invitrogen, UK). PCR primers specific to the eEF1A1 isoform were designed manually, using the Ensembl cDNA sequence: ENST00000316292 (http://www.ensembl.org/index.html). The eEF1A1-forward primer sequence was (5′-3′): TCCTTCAAGTATGCCTGGGTCT (eEF1A1-F1), corresponding to nucleotide positions 157–178. The eEF1A1-reverse primer sequence was: TGGCACAAATGCTACTGTGTCG (eEF1A2-R1), corresponding to nucleotide positions 555–576, to give an expected PCR product size of 420 bp. Similarly PCR primers specific for the eEF1A2 isoform were designed using the Ensembl cDNA sequence: ENST00000298049. The eEF1A2-forward primer sequence was: AGGAGGCTGCTCAGTTCACCT (eEF1A2-F3), corresponding to nucleotide positions 1004–1024; and the eEF1A2- reverse primer sequence was: CCGCTCTTCTTCTCCACGTTC (eEF1A2-R3), corresponding to nucleotide positions 1317–1336, with an expected PCR product size of 334 bp. Primers were synthesized using the commercial facility at Eurofins MWG Operon (http://www.eurofinsdna.com/products-services/oligonucleotides0.html).

Reverse transcription PCR was performed by using 1 µl of cDNA from each of the cell lines, 10 pmol of each forward/reverse primer, and 0.5 µl of AccuPrime Taq DNA polymerase (Invitrogen, UK), in 20 µl volumes. Thermocycling was performed under the following conditions: Initial denaturation at 94°C for 5 minutes; 30 PCR cycles of 94°C for 1 min, 58°C for 1 min, and 72°C for 1 min, and a final extension of 72°C for 7 minutes. Amplified PCR products (10 µl) were separated on a 2.5% agarose gel containing ethidium bromide and imaged using the GelDoc XR^+^ Molecular Imager (Bio-Rad). Band intensities were measured using the Quantity One software (Bio-Rad).

PCR products were sequenced at the Genetics core facility, University of Sheffield (http://www.shef.ac.uk/medicine/research/corefacilities/genetics.html). DNA sequences were visualised using the Chromas Lite version 2.01 software, freely downloaded from http://www.technelysium.com.au/chromas_lite.html.

## Results

### Hierarchical cluster analysis of protein profiles

**Figure 1:**
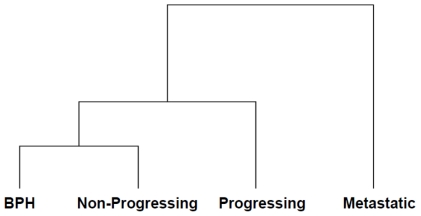
Hierarchical cluster analysis of the 4 patient groups studied. Samples were clustered based on the similarity of their protein expression profiles observed in log_10_ of the iTRAQ ratios and a dendrogram generated to indicate the relationship between the samples. Squared Euclidean distance between clusters (single linkage) is shown. Varying lengths of the branch points indicate the degree of similarity; the shorter the branch the higher the degree of similarity.

Analysis of the 4 pooled groups of patients identified 122 proteins with associated information on their relative expression levels (Table S2). The false discovery rate was 1.4% which is within the acceptable limit of 5% [25]. For the iTRAQ dataset, 75 unique proteins were identified and relatively quantified with ≥2 unique peptides, and these data were used for statistical analyses to determine alterations in protein levels between groups. Hierarchical clustering was performed to group the data based on the degree of similarity between the BPH and cancer groups. Agglomerative clustering using the squared Euclidean distance between log_10_ value of iTRAQ ratios and smallest intercluster dissimilarity linkage procedure was performed (Mathematica 7.0.0 for Mac), to generate the dendrogram shown in Figure 1. A key feature of the dendrogram is separation of the patient groups according to the stage of their disease. Patients with metastasis separated the furthest and are thus considered to be the most different from patients with BPH. The patients with BPH clustered more closely with patients in the non-progressing group compared with the progressing and metastatic groups.

### Gene ontology annotation

To assess the range of proteins identified, gene ontology (GO) annotations for biological processes were assigned using the Protein ANalysis THrough Evolutionary Relationships (PANTHER) software, which links protein accession codes to the corresponding entries in the gene ontology database [14], [27]. The PANTHER analysis revealed the presence of many common plasma proteins such as those associated with complement mediated immunity (14%), immunity and defence (27%), proteolysis (21%), blood clotting (7%), and protein metabolism and modification (22%).

For the biological network analysis, the Metacore platform (GeneGo, Inc., St. Joseph, MI), was employed as previously described [14], which revealed that many of the differentially expressed proteins such as C4, C4a, C5, C5b, C9 and C6 mapped to the classical immune response pathway (Figure S1).

### Diagnostic biomarker leads

Differences in protein levels are reported following a t-test analysis as previously described [23]. The p-values were calculated based on the number of peptides used for the quantification and the variance of the iTRAQ reporter ratios derived from these peptides. A p-value≤0.01 was used to assign significant changes in protein levels between sample sets. Importantly, the protein changes reported as significant differential expression were selected based upon statistical significance rather than fold change [28]. Some of these differences are expected to be potentially larger due to the known under estimation associated with iTRAQ based quantification [24].

During our analysis we were interested in proteins showing both increased and decreased expression levels, as previous studies have reported that both significantly up- and down-regulated proteins may be of clinical relevance [15], [29].

The identification of proteins differentially expressed in non-progressing, progressing and metastatic patient groups relative to the BPH group were of interest as these could provide leads for potentially useful diagnostic and prognostic biomarkers (Table S3). Thus, a comparison between the non-progressing cancer group versus the BPH group showed a significant differential expression of 22 proteins; 7 of which were up-regulated and 15 down-regulated (Table S3a). Similarly, a comparison between the progressing patient group versus the BPH group identified the differential expression of 19 proteins; 11 of which showed significant over-expression and 8 showed down-regulation (Table S3b). Comparisons of the metastatic patient group versus the BPH group identified the differential expression of 35 proteins, with 19 proteins showing significant over-expression and 16 showing significant down-regulation (Table S3c). Additionally, C-reactive protein (CRP) was found to be elevated 41.1 fold in the serum of patients with metastatic disease compared to patients with BPH (Table S2).

### Prognostic biomarker leads

Once a diagnosis of cancer has been made the next steps are to establish the extent (stage) of disease, in an attempt to predict those patients in which the disease is likely to progress from the patients in which the disease is likely to remain localized, and to obtain prognostic information. Currently, pre-treatment PSA levels, biopsy Gleason grade and clinical staging are used to provide prognostic information; however, these parameters are associated with a number of limitations. Thus, a comparison of patients with progressing versus non-progressing disease identified the significant differential expression of 25 proteins; 13 up-regulated and 12 down-regulated (Table S4).

### Differential protein levels associated with disease progression

**Figure 2:**
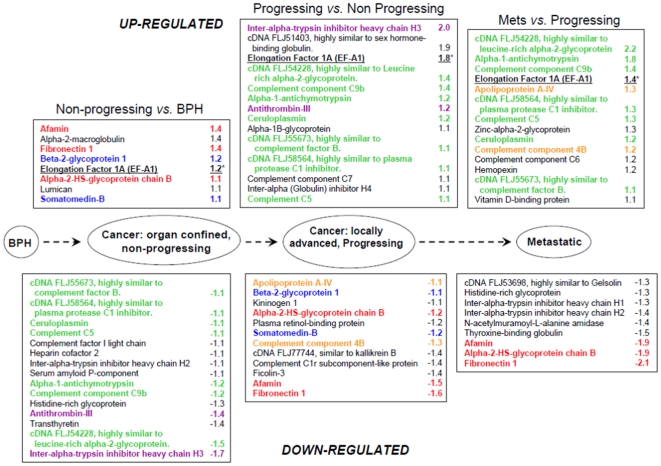
Proteins showing significant differential expression (up-regulated and down-regulated) according to disease progression. The list of differentially expressed proteins shown are based on comparisons between non-progressing versus BPH; progressing versus non-progressing and metastatic versus progressing groups. Note the differential expression of proteins either individually (black font), as pairs (blue, orange and purple fonts), or as a panel (≥3 proteins, green and red fonts). (*) = identified as a single high confidence peptide.

In addition to the comparisons above, protein differences were mapped according to the stage of prostate cancer development and progression i.e. as the cancer developed from non-malignant epithelium and progressed to locally advanced and metastatic disease (Figure 2). The lists of differences are based on comparisons between the non-progressing versus BPH group; progressing versus non-progressing group; and metastatic versus progressing cancer groups. From Figure 2, it is apparent that individual proteins, ‘pairs’ of proteins and ‘panels’ of proteins (defined as ≥3 proteins), were seen to be differentially expressed at certain stages of disease development and progression. For instance, individual proteins such as alpha-2-macroglobulin, lumican and serum amyloid P-component were seen to be differentially expressed between the non-progressing versus the BPH group. Other proteins such as beta-2-glycoprotein 1 and somatomedin-B (blue font), were both seen to be relatively decreased as a ‘pair’ in progressing versus non-progressing samples, while both Apolipoprotein A-1V and Complement component 4B were seen to be relatively increased in expression in metastatic samples versus progressing samples (orange font). Additionally, two ‘panels’, were seen to be altered as the disease developed and progressed. The first panel comprised of 3 proteins: afamin, alpha-2-HS-glycoprotein chain B and fibronectin 1 (shown in red font), and was seen to be relatively increased in expression in the non-progressing versus the BPH group, but decreased as the cancer progressed and remained relatively low as the cancer metastasized. The second panel comprised of 7 proteins: alpha-1-antichymotrypsin; cDNA FLJ55673, highly similar to complement factor B; cDNA FLJ54228, highly similar to leucine-rich alpha-2-glycoprotein; cDNA FLJ58564, highly similar to plasma protease C1 inhibitor; Ceruloplasmin, Complement C5 and Complement component C9b (green font), and were seen to be relatively decreased in expression in the non-progressing group compared with the BPH group, and relatively increased in expression as the cancer progressed i.e. were relatively increased in the progressing group and remained elevated in the metastatic group.

Interestingly, eukaryotic translation elongation factor 1 alpha 1 (eEF1A1), (a.k.a EF-Tu), was seen to show significant increased expression in non-progressing cancer relative to BPH, and its expression was further increased with disease progression, and was maintained during metastasis (Table S2 and Figure 2). eEF1A1 was the top hit following the blastp search of the VETGVLKPGMVVTFAPVNVTTEVK peptide identified in the serum samples. Comparison of the full length amino acid sequence of eEF1A1 with its isoform eEF1A2, indicated that the peptide sequence was unique to eEF1A1. The corresponding peptide in eEF1A2 differs by a single amino acid where valine is substituted by isoleucine. Since these amino acids have a 14 Da difference in molecular mass we could confidently assign the identified peptide to correspond to the eEF1A1 isoform.

### Confirmation of candidates identified by iTRAQ

To confirm the differential expression of candidate proteins identified by iTRAQ, we subsequently performed a 1D-gel electrophoresis using pooled serum from the 4 patient groups. Following staining with Coomassie blue, a faint band was visually seen to be present at a slightly higher intensity in the samples from patients with metastatic disease relative to the other 3 groups of patients (data not shown). This band was excised from the gel, digested with trypsin and the resultant peptides analysed by LC-MS/MS. The mass spectrometry data identified 7 peptides matching to CRP with a sequence coverage of 27.6%, and confirmed the iTRAQ data. The mass spectrum of a representative iTRAQ labelled peptide from CRP protein following MS/MS is shown in Figure S2.

### eEF1A1 immuno-expression in prostatic tissue

During our iTRAQ analysis, we identified eEF1A1 to be increased in expression in all of the cancer groups relative to BPH, with relatively higher levels seen in the progressing (+1.8 fold), and metastatic groups (+1.4 fold), (Table S2, and Figure 2). eEF1A1 was of particular interest to us for a number of reasons. Our previously published iTRAQ study had shown its levels to be increased in higher metastatic variant prostate cancer cells [14]. Furthermore, a previous study had shown that down-regulation of eEF1A1 by RNA interference (RNAi), in Du145 cells reduced cell proliferation, and inhibited cell migration and invasion [30].

**Figure 3:**
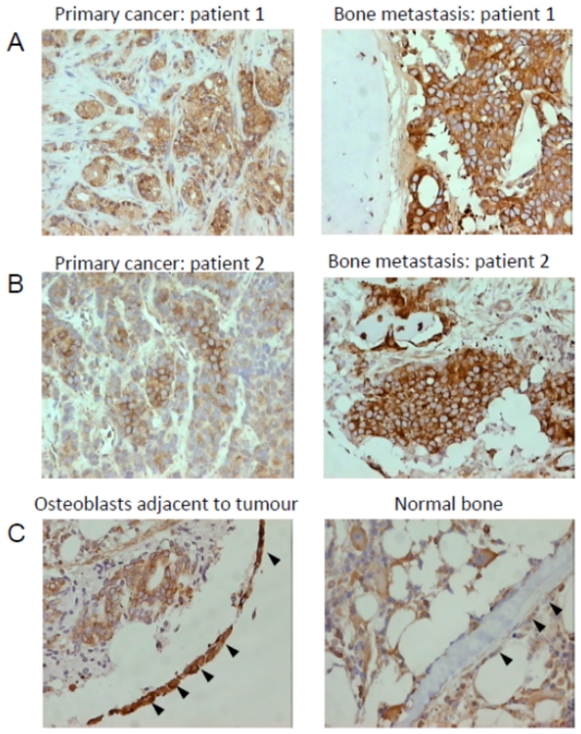
Representative images showing the immunoexpression of eEF1A1 in prostatic tissue, bone metastatic lesions and control bone. (A), eEF1A1 immunoexpression can be seen in the malignant cells of the primary cancer (staining intensity = 2), and in the matched bone metastatic lesion from Patient 1 (staining intensity = 3). (B), eEF1A1 immunoexpression in the malignant cells of the primary cancer (staining intensity = 1), and in the matched bone metastatic lesion from Patient 2 (staining intensity = 3). (C), Intense eEF1A1 immunoexpression can be seen in the osteoblasts lining bone (staining intensity = 3, *arrowhead*s), which are in close proximity to tumour cells, whereas osteoblasts lining normal bone show weak expression (staining intensity = 1, *arrow heads*).

Thus, immunohistochemical (IHC) staining for eEF1A1 was performed using clinical tissue material from patients with BPH, organ confined cancer, and bone from patients both with and without metastatic prostate cancer. Representative immunostaining images are shown in Figures 3A–C. Although eEF1A1 immunoexpression was seen in cancer cells, there was no significant difference in the staining intensity between organ confined disease and metastatic disease (p = 0.1720, Mann Whitney U). Interestingly, intense immunoexpression of eEF1A1 was seen in osteoblasts and in particular those osteoblasts in the vicinity of metastatic prostate tumour cells (Figure 3C). Assessment of the immunostaining intensity in bone metastatic lesions showed that there was a statistically significant higher median immunostaining intensity in the osteoblasts adjacent to metastatic prostate cancer cells (n = 6 cases, median = 2.0), compared to osteoblasts in normal control bone samples (n = 15 cases, median = 0), (p = 0.0353, Mann Whitney U).

### Prostate cancer cell lines express both the eEF1A1 and eEF1A2 isoforms

**Figure 4:**
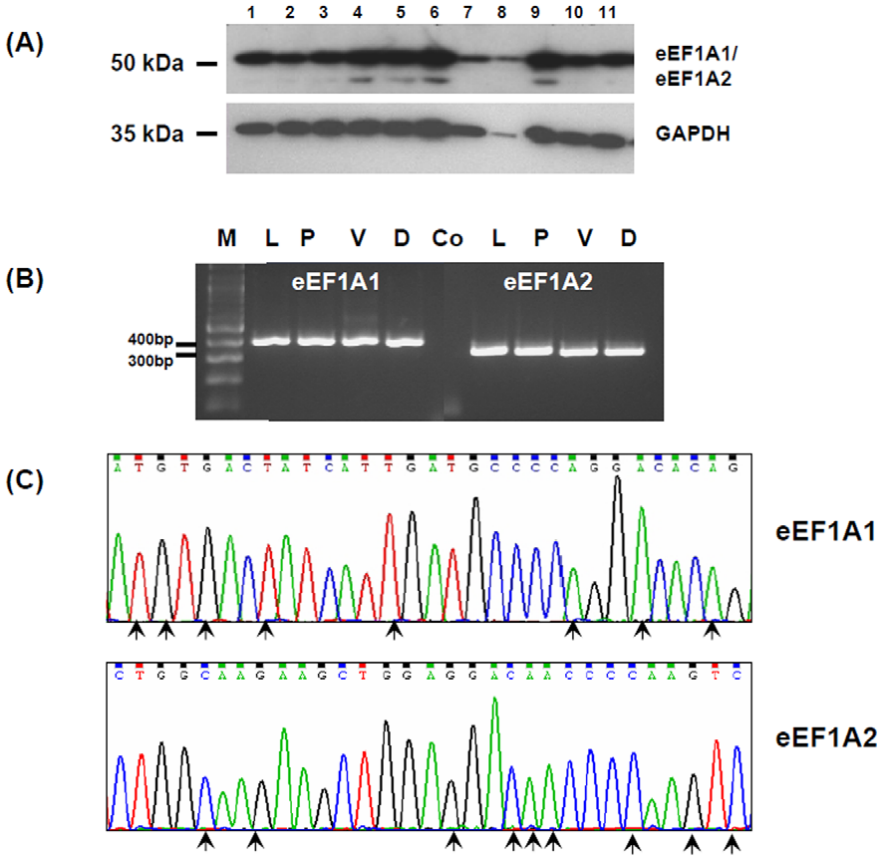
The expression of eEF1A1 and eEF1A2 isoforms in human prostate cancer cell lines. (A), Western blotting performed on prostate cancer cell lines using an antibody reactive to both the eEF1A1 and eEF1A2 isoforms. GAPDH was used as a loading control. Twenty-five micrograms of protein were loaded in each lane. Lanes 1–11: 1 = LNCaP; 2 = LNCaP-LN3; 3 = LNCaP-Pro5; 4 = LNCaP-C42; 5 = LNCaP-C4-2B; 6 = DuCaP; 7 = VCaP; 8 = Du145; 9 = PC-3; 10 = PC-3M; 11 = PC-3M-LN4. (B), Reverse-transcription PCR specific for the eEF1A1 and eEF1A2 isoforms, performed using mRNA extracted from prostate cancer cell lines: L = LNCaP; P = PC-3; V = VCaP; D = DuCaP. Control (Co), PCR was performed without mRNA. Relative equal expression of eEF1A1 and eEF1A2 mRNA can be seen in all cell lines tested (i.e. relative intensity ratio of 1.0, using Quantity One). (C), Sections of the DNA sequence chromatograms generated by sequencing the PCR products from the LNCaP cell line, confirming the specificity of the PCR primers used. Nucleotide bases unique to each isoform are marked by arrows.

eEF1A occurs as two isoforms i.e. eEF1A1 and eEF1A2 with the proteins sharing 92% sequence identity (http://omim.org/entry/602959). In order to investigate the expression of both isoforms we performed Western blotting using 11 human prostate cancer cell lines and an osteosarcoma cell line (TE-85), with an antibody directed against the N-terminus of both isoforms. As expected a single immunoreactive band was detected at ∼50 kDa in all cell lines tested (Figure 4A), and in the osteosarcoma cell line (data not shown). To investigate further the relative expression of the two isoforms, we performed semi-quantitative reverse transcription PCR using mRNA extracted from LNCaP, PC-3, VCaP and DuCaP prostate cancer cell lines with isoform specific primers. The expression of both the eEF1A1 and eEF1A2 isoforms was detected in all 3 cell lines tested with a relative equal intensity i.e. an expression ratio of 1.0 (Quantity One software, Bio-Rad), (Figure 4B) . The specificity of the PCR primers was confirmed by sequencing the PCR products (Figure 4C).

## Discussion

In an effort to identify leads for potentially useful serum biomarkers for prostate cancer diagnosis and progression, we profiled pooled serum samples from 4 carefully selected groups of patients representing the various stages of prostate cancer development and progression using a 4-plex iTRAQ approach. Following the GO annotations of the 75 proteins identified and quantified (≥2 peptides), the majority of these were found to class to diverse biological pathways such as protein metabolism and modification; blood clotting; proteolysis; immunity and defence; complement mediated immunity; blood circulation and gas exchange. Regarding the differentially expressed proteins, some of these have previously been reported as candidate prostate cancer biomarkers such as CRP, alpha-2-macroglobulin, ceruloplasmin, zinc-alpha-2-glycoprotein, beta-2-microglobulin and fibronectin, which provides confidence to our dataset and provides an independent confirmation of these candidates [31]–[34].

Of the proteins previously associated with prostate cancer; CRP is an acute phase reactant (APR) protein produced by the liver in response to inflammation. Elevated levels of CRP have been reported in patients with bone metastatic prostate cancer, and have been associated with an adverse outcome for men with castration resistant prostate cancer [32]. Thus, our finding of elevated levels of CRP in metastatic cases is consistent with previous studies. In addition to CRP, many other proteins seen to be differentially expressed between patient groups were found to class to the APR protein family and include fibrinogen, alpha-2-macroglobulin, ceruloplasmin, haptoglobin, alpha-1- acid glycoprotein, alpha-1-antitrypsin and alpha-1-antichymotrypsin (Figure 2). Pathway analysis of the differentially expressed proteins showed that a number of these such as C4, C4a, C5, C5b, C9 and C6 mapped to the classical inflammatory pathway (Figure S1). Although this finding is perhaps not surprising as it is well documented that the presence of a tumour activates an inflammatory response, another possibility is that at least some of these APR proteins could have been secreted ‘ectopically’ by the tumour cells themselves. In support of this possibility, previous studies have reported that renal cell carcinoma, squamous cell carcinoma and breast cancer cell lines may produce and secrete common plasma proteins such as albumin, prealbumin, alpha-1-antitrypsin, ceruloplasmin, alpha-2-macroglobulin, haptoglobin, transferrin and alpha-1-antichymotrypsin [35], [36]. Furthermore, the potential of assessing APR proteins as cancer biomarkers has already been reported in a previous study showing that levels of APR proteins in patients with prostate cancer could aid diagnosis and staging, and allowed the correct identification of metastatic disease in 88.6% of patients [37]. Thus, our data support the possibility of assaying a combination of APR proteins secreted by the tumour cells themselves, as well as APR proteins produced by the liver during an immune response. Although, the detection of APR proteins in serum may provide valuable diagnostic/prognostic information these proteins could also potentially hamper the identification of *bona fide* cancer specific biomarkers due to their relatively higher abundance in the serum of cancer patients. For instance it has been shown that circulating concentrations of serum amyloid A are transiently increased as much as 1000-fold in response to inflammation [38]. Thus, any future biomarker discovery programmes may additionally benefit from the prior removal of major APR proteins in an effort to improve the detection of relatively lower abundance biomarkers.

Another candidate identified as being relatively up-regulated in metastatic cases compared with the other groups was Beta-2-microglobulin (B2M), (Table S2). B2M is a component of the MHC I complex and has been shown to be released by LNCaP prostate cancer cells in culture in response to androgen stimulation [39]. There have been a number of reports implicating B2M as a candidate prostate cancer biomarker. For instance, elevated levels have been detected in prostatic secretions of patients with metastatic prostate cancer [40]. Serum B2M levels have been shown to be elevated in patients with metastatic, androgen-independent prostate cancer [31]. Additionally, B2M has functionally been implicated in prostate cancer as its over-expression in cancer cells induced rapid tumour growth in bone, while disrupting B2M signaling by specific small interfering RNA produced a regression of previously established prostate tumours [41]. Thus, B2M may potentially serve as a biomarker for prostate cancer progression and a novel drug target for the treatment of bone metastasis which requires further study.

Other candidates up-regulated in one or more of the cancer groups compared with the BPH group were fibronectin 1, afamin, alpha-2-HS-glycoprotein chain B, ceruloplasmin and beta-2-glycoprotein 1 (Figure 2). Interestingly, fibronectin was very recently shown to be amongst the five-protein signature panel with potential for Gleason score prediction [34]. Furthermore, in a recent study fibronectin has been shown to be involved in initiating lung cancer metastasis [42], thus making fibronectin an attractive candidate biomarker and therapeutic target.

Another promising candidate identified in our study was Afamin, which was found to be up-regulated in the non-progressing group compared with BPH group, but was down-regulated in the progressing and metastatic disease (Figure 2). Afamin is a member of the albumin gene family expressed by the liver and kidneys, and has been shown to be a specific binding protein for vitamin E. Interestingly, our data is consistent with a previous study which showed a decrease in afamin levels in ovarian cancer where it has been proposed as a candidate biomarker [43]. Thus, the potential of afamin as a candidate prostate cancer biomarker requires further study.

eEF1A1, is a member of the elongation factor proteins that normally functions to mediate the selection and binding of the aminoacyl-tRNA to the ribosome during protein synthesis, and ensures translational accuracy. eEF1A1 was of particular interest to us as we had previously shown its levels to be increased in higher metastatic variant prostate cancer cells [14], and another study had reported that down-regulation of eEF1A1 by RNA interference (RNAi) in Du145 cells led to an inhibition of cell proliferation, invasion and migration [30]. Interestingly a truncated form of eEF1A1 known as prostate tumour inducing gene 1 (PTI-1) has been shown to be expressed in prostate carcinoma patient-derived blood samples and proposed to be a sensitive biomarker for prostate cancer [44]. Additionally, PTI-1 was shown to be expressed in prostate cancer tissues but not in BPH or normal prostate tissues [45]. Our finding of increased eEF1A1 expression in osteoblasts in the vicinity of metastatic prostate cells, is in line with previous reports indicating a cross-talk between prostate cancer cells and osteoblasts during bone metastasis [46]. Prostate cancer bone metastasis are typically osteoblastic (i.e. involve excessive bone formation), and it is well known that tumour cells can stimulate osteoblasts to proliferate and differentiate. Thus, eEF1A1 over-expression seen in osteoblasts may occur as a response to the presence of tumour cells. The increased expression of eEF1A1 in the serum of patients with cancer compared with BPH is an important finding as is suggests that the search for novel cancer biomarkers should encompass not only those factors secreted directly by the cancer cells but also ‘surrogate markers’ produced indirectly as a reaction to the presence of tumour cells.

During our analysis we identified and quantified 122 unique proteins (of which 75 were qualified by ≥2 unique peptides). This is in line with a previous iTRAQ study analysing serum samples with similar MS instrumentation [47]. Although depletion strategies can improve sensitivity for detection of less abundant proteins, this approach itself is associated with limitations as the higher abundant proteins are known to act as a ‘sponge’ by complexing with lower molecular weight proteins. For instance, a targeted analysis of alpha-2-macroglobulin binding partners indicated that this highly abundant plasma glycoprotein can bind various cytokines, growth factors and heat shock proteins [48]. Consistent with this, we were unable to detect PSA during our iTRAQ analysis despite it being known to be present at relatively high concentrations in the cancer and metastatic patient groups. PSA is known to complex with alpha-1-antitrypsin and alpha-2-macroglobulin, both of which were targets for removal by the IgY-14 affinity column [49]. Thus, it is important that future biomarker studies analyze both the bound and flow-through fractions in order to increase the repertoire of disease candidates identified.

Although our study identified the differential expression of a number of proteins, it is becoming apparent that a single biomarker is unlikely to provide the required sensitivity and specificity, due to the heterogeneity and dynamic nature of prostate cancer [50]. Thus, it has been proposed that accurate disease diagnosis and prognosis are likely to depend on the measurement of a panel of biomarkers, perhaps utilising emerging multiplex technologies which could fast forward these panels into the clinic. In support of this, our data has shown that levels of certain combinations of proteins could be seen to fluctuate as the disease developed and progressed. Certain proteins were seen to be differentially expressed either individually, as pairs or as panels (Figure 2). Interestingly, a previous study has reported that by using a combination of proteins (complement component 4a (C4a) and protein C inhibitor), a statistically significant value for predicting prostate cancer recurrence was demonstrated in men who underwent prostatectomy [51]. Thus, our study adds weight to other published studies demonstrating the importance of evaluating pairs or panels of carefully selected proteins to increase the diagnostic and prognostic accuracy of cancer.

To our knowledge, our study appears to be the first iTRAQ based approach aimed at identifying leads for potentially useful biomarkers of progression and metastasis in prostate cancer, using patient serum. If the findings are validated in a larger cohort of patients, then the detection of eEF1A1 in the serum of patients with prostate cancer at the time of their initial diagnosis, may be able to predict the likelihood of disease progression and those patients be offered radical treatment options at an early stage of their disease.

With a number of biomarker studies already conducted over the years and others in the pipeline, what is becoming evident is that in order for a biomarker (or panel of biomarkers) to reach routine clinical use it must pass through five phases of development [52], [53]. Thus, our study represents one of the initial steps (‘exploratory phase’) along this process. Many of the candidates identified in our and other studies await rigorous clinical validation using large cohorts of patient samples, together with robust long-term clinical and pathological information in subsequent phase 2–5 studies, as proposed by Pepe *et al.*, [52], [53]. In addition to the leads identified, what has emerged is that due to the sizable inflammatory response to the presence of a tumour, future biomarker identification strategies could benefit by the pre-fractionation of APR proteins from the sera of cancer patients in addition to the removal of common highly abundant serum proteins, so that the cancer proteome can be mined even more deeply. The panel of proteins identified, including eEF1A1 warrant further investigation and validation.

## Supporting Information

Figure S1 **Metacore pathway analysis of proteins differentially expressed between metastatic and progressing patient groups, showing proteins mapping to the classical immune response pathway.** Proteins shown with a red thermometer symbol represent increased expression levels.
(TIF)

Figure S2 **Representative tandem mass spectra for C-reactive protein and insert showing the peak area at the low mass/charge (m/z) region with the iTRAQ reporter ions.**
(TIF)

Table S1 **Disease characteristics of the 20 patients comprising the 4 groups of patients analysed by iTRAQ.**
(DOC)

Table S2 **Full list of the 122 proteins identified by iTRAQ.**
(RTF)

Table S3 **Proteins differentially expressed between various cancer groups and BPH.** (S3a) Proteins differentially expressed between the non-progressing and BPH group. (S3b), proteins differentially expressed between the progressing and BPH group. (S3c), proteins differentially expressed between the metastasis and BPH group.
(RTF)

Table S4 **Proteins differentially expressed between the progressing versus non-progressing group.**
(RTF)
