# Correction: iTRAQ Identification of Candidate Serum Biomarkers Associated with Metastatic Progression of Human Prostate Cancer

**DOI:** 10.1371/annotation/d54d7c5b-ed36-43bb-a888-65cc7061ed09

**Published:** 2012-05-25

**Authors:** Ishtiaq Rehman, Caroline A. Evans, Adam Glen, Simon S. Cross, Colby L. Eaton, Jenny Down, Giancarlo Pesce, Joshua T. Phillips, Saw Yen Ow, George N. Thalmann, Phillip C. Wright, Freddie C. Hamdy

The tenth author's name was spelled incorrectly. The correct name is: Saw Yen Ow.

