# Supplementary figures and images for: iTRAQ Identification of Candidate Serum Biomarkers Associated with Metastatic Progression of Human Prostate Cancer
Source: PLoS One. 2012 Feb 15;7(2):e30885. doi: 10.1371/journal.pone.0030885 (PMC3280251; doi:10.1371/journal.pone.0030885)

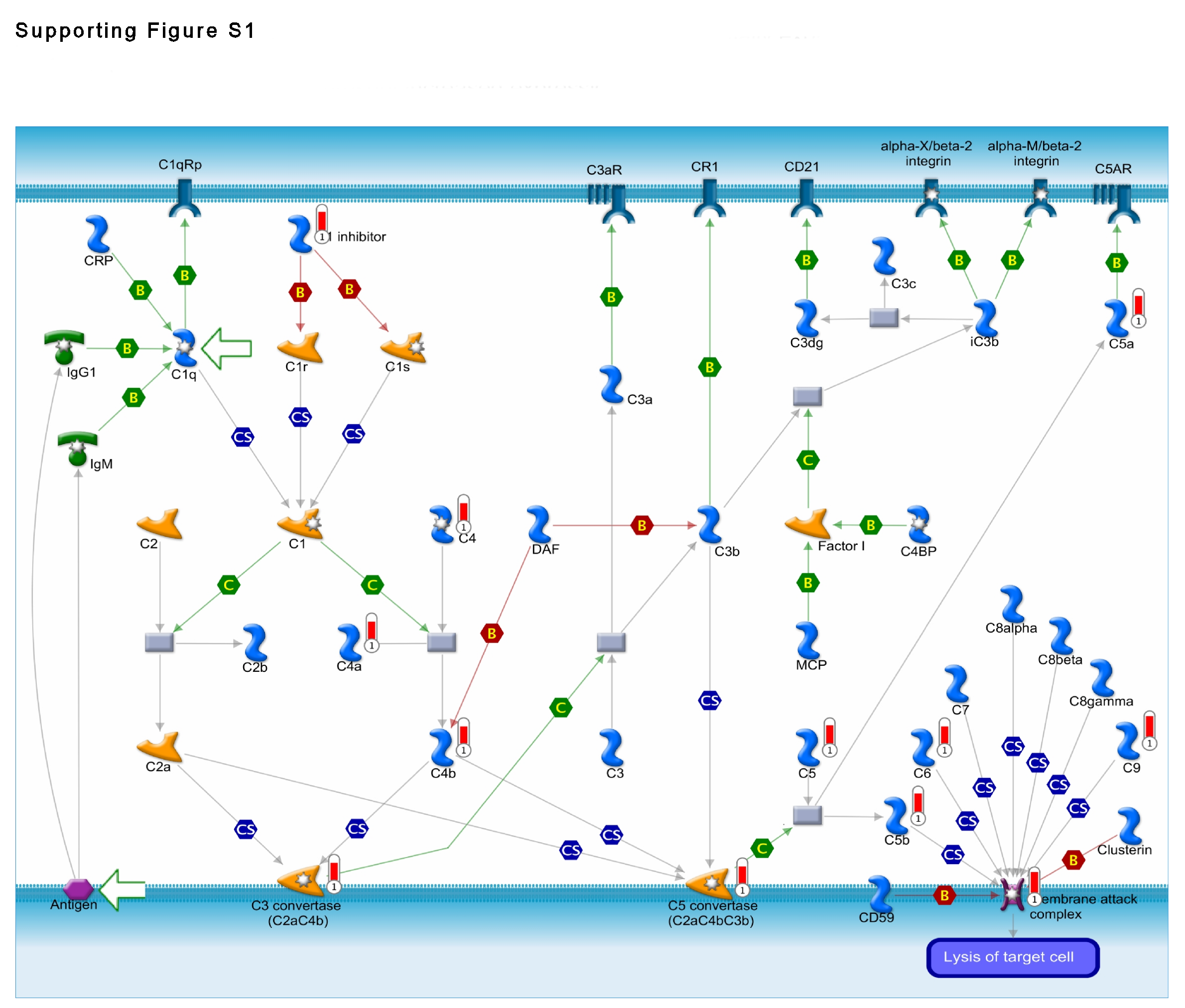

Supplement: Figure S1 — Metacore pathway analysis of proteins differentially expressed between metastatic and progressing patient groups, showing proteins mapping to the classical immune response pathway. Proteins shown with a red thermometer symbol represent increased expression levels. (TIF) [file pone.0030885.s001.tif]

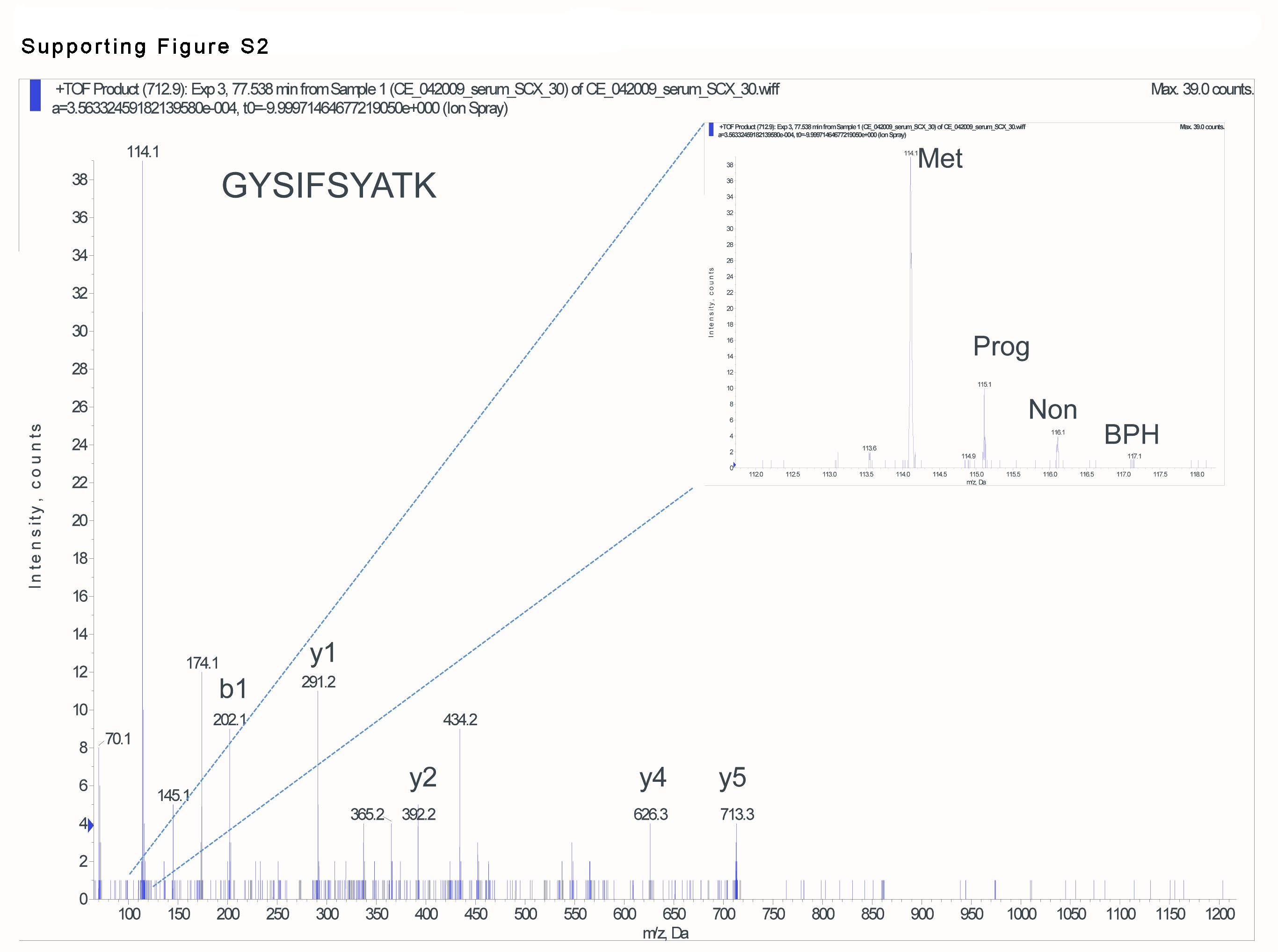

Supplement: Figure S2 — Representative tandem mass spectra for C-reactive protein and insert showing the peak area at the low mass/charge (m/z) region with the iTRAQ reporter ions. (TIF) [file pone.0030885.s002.tif]
