# Supplementary material for: iTRAQ Identification of Candidate Serum Biomarkers Associated with Metastatic Progression of Human Prostate Cancer
Source: PLoS One. 2012 Feb 15;7(2):e30885. doi: 10.1371/journal.pone.0030885 (PMC3280251; doi:10.1371/journal.pone.0030885)
Supplement: Table S1 — Disease characteristics of the 20 patients comprising the 4 groups of patients analysed by iTRAQ. (DOC) [file pone.0030885.s003.doc]

**Table S1.** Disease characteristics of the 20 patients comprising the 4 groups of patients analysed by iTRAQ.

| **Patient** | **Age/**  **yrs §** | **Group¶** | **Initial**  **stage** | **Initial**  **PSA**  **(ng/ml)** | **Histology** | **Management#** | **Bone**  **scan** |
| --- | --- | --- | --- | --- | --- | --- | --- |
| 312 | 64 | BPH | n/a | 3.6 | 2 negative biopsies | No treatment | n/a |
| 314 | 69 | BPH | n/a | 2.7 | 2 negative biopsies | No treatment | n/a |
| 315 | 60 | BPH | n/a | 6.1 | 2 negative biopsies | No treatment | n/a |
| 367 | 60 | BPH | n/a | 3.7 | 2 negative biopsies | No treatment | n/a |
| 368 | 50 | BPH | n/a | 3.5 | 2 negative biopsies | No treatment | n/a |
| 204 | 71 | Non-prog. | T1c | 3.3 | Gleason score 6 (3+3) | Active monitoring | n/a |
| 323 | 60 | Non-prog | T1/2 | 8.3 | Gleason score 6 (3+3) | Active monitoring | n/a |
| 346 | 64 | Non-prog | T1c | 3.1 | Gleason score 6 (3+3) | Active monitoring | n/a |
| 354 | 71 | Non-prog | T2a | 2.1 | Gleason score 6 (3+3) | Active monitoring | n/a |
| 360 | 67 | Non-prog | T1/2 | 4.3 | Gleason score 6 (3+3) | Active monitoring | n/a |
| 5 | 72 | Prog | T1/2 | 5.1 | Gleason score 6 (3+3) | Active monitoring | No mets |
| 167 | 70 | Prog | T1/2 | 5.9 | Gleason score 6 (3+3) | Active monitoring | No mets |
| 192 | 69 | Prog | T1/2 | 10.0 | Gleason score 6 (3+3) | Active monitoring | No mets |
| 227 | 67 | Prog | T1/2 | 8.9 | Gleason score 6 (3+3) | Active monitoring | No mets |
| 460 | 67 | Prog | T1/2 | 12.2 | Gleason score 6 (3+3) | Active monitoring | No mets |
| 182 | 80 | Mets | T3/4 | 65.1 | Gleason score 6 (3+3) | Hormones+ BSO | Mets |
| 308 | 65 | Mets | T3 | 19.3 | Gleason score 9 (4+5) | Casodex, BSO, | Mets |
| 362 | 53 | Mets | T4 | 159 | Gleason score 8 (3+5) | Hormones | Mets |
| 431 | 82 | Mets | T3 | 188 | Gleason score 9 (4+5) | BSO | Mets |
| 444 | 86 | Mets | T4 | 25.5 | Gleason score 9 (5+4) | BSO | Mets |

**Footnotes:** Abbreviations: n/a = not applicable; Mets = metastasis; BSO = Bisphosphonates; BPH = benign prostatic hyperplasia; Prog = progressing. Patient groups were (i) BPH (ii) non-progressing (ii) progressing (iv) metastatic. **§** Age at the time of blood sampling. **¶** BPH patients were those with a histological diagnosis of BPH following at least 2 sets of prostatic biopsies. Non-progressing group comprised of patients with organ confined prostate cancer diagnosed by histological assessment of prostatic biopsies, and a PSA level less than 10ng/ml with no significant changes in serum PSA levels during 5 years of active monitoring. Progressing group of patients had organ confined cancer at initial presentation by histological assessment of prostatic biopsies, a initial PSA level below 13ng/ml, but had 3 consecutive rises in PSA levels during 5 years of active monitoring. Metastatic patients were those with confirmed bone metastasis at presentation as determined by radionucleotide bone scan. **#** Patient management subsequent to blood sampling.
