# Supplementary material for: iTRAQ Identification of Candidate Serum Biomarkers Associated with Metastatic Progression of Human Prostate Cancer
Source: PLoS One. 2012 Feb 15;7(2):e30885. doi: 10.1371/journal.pone.0030885 (PMC3280251; doi:10.1371/journal.pone.0030885)
Supplement: Table S2 — Full list of the 122 proteins identified by iTRAQ. (RTF) [file pone.0030885.s004.rtf]

Table S2. Full list of the 122 proteins identified by iTRAQ.

Score	#valid pept sequences	% Cov	pI	Mass Da	Acccession number	Protein name	# peptides for quant	Mets	Prog	Non-Prog	BPH	
168.9	18	34.9	6.0	70586	Q14624	Inter-alpha-trypsin inhibitor heavy chain H4 	4	1.2	1.2	1.0	1	
7.4	1	3.5	5.6	24544	Q15848	Adiponectin 	1	1.3	1.3	0.8	1	
162.3	18	32.2	5.6	66577	P43652	Afamin	53	0.5	0.9	1.4	1	
166.2	16	43.8	5.4	45266	P01011	Alpha-1-antichymotrypsin His-Pro-less 	12	1.8	1.1	0.8	1	
168.6	16	41.8	5.7	51941	P04217	Alpha-1B-glycoprotein 	129	1.1	1.1	1.0	1	
77.4	8	27.7	6.0	50451	P08697	Alpha-2-antiplasmin 	19	0.9	1.0	1.1	1	
68.2	6	22.9	5.5	39325	P02765	Alpha-2-HS-glycoprotein chain B	68	0.5	0.9	1.1	1	
24.2	3	2.6	6.0	160811	P01023	Alpha-2-macroglobulin (Alpha-2-M) 	4	1.2	1.9	1.4	1	
7.7	1	6.4	7.7	12943	P07477	Alpha-trypsin chain 2 	2	1.0	0.8	1.0	1	
6.5	1	4.6	6.0	24119	Q01432A	AMP deaminase 3 	1	1.8	1.7	2.4	1	
110.2	11	31.6	5.7	49761	P01019	Angiotensin-3 (Ang III) 	70	1.3	1.1	1.0	1	
168.1	18	46.1	6.1	49039	P01008	Antithrombin-III 	93	1.0	0.9	0.7	1	
240.5	25	69.9	5.2	43403	P06727	Apolipoprotein A-IV 	153	1.2	0.9	1.0	1	
134.4	14	50.6	8.5	36255	P02749	Beta-2-glycoprotein 1 (Apo-H) 	87	1.2	1.1	1.2	1	
8.7	1	10.2	6.5	11618	P61769	Beta-2-microglobulin 	2	1.4	0.8	1.0	1	
27.4	3	9.4	5.1	53864	Q96KN2	Beta-Ala-His dipeptidase 	4	0.8	0.7	1.0	1	
25.8	3	6.6	5.4	56772	P43251	Biotinidase 	4	0.7	1.0	1.1	1	
16.1	2	11.5	5.0	26351	P20851	C4b-binding protein beta chain 	2	2.8	2.4	2.6	1	
6.6	1	1.7	6.7	54088	Q7LDD5	Calcium/calmodulin-dependent protein kinase 	1	1.5	0.8	0.7	1	
15.3	2	6.1	8.4	35788	Q96IY4	Carboxypeptidase B2 	4	1.1	1.2	0.8	1	
23.1	3	7.8	7.1	50034	P15169	Carboxypeptidase N catalytic chain	6	1.0	0.9	0.8	1	
53.3	6	15.5	5.6	58285	P22792	Carboxypeptidase N subunit 2 	12	1.1	0.9	1.0	1	
6.9	1	27.3	6.9	4852	C1PHC4	CD44 molecule	2	0.7	1.5	0.7	1	
6.6	1	5.6	11.6	23734	Q6ZMZ4	cDNA FLJ16563 fis, clone SYNOV4005739	1	0.1	0.0	0.7	1	
6.9	1	3.4	5.1	25469	B7Z8T3	cDNA FLJ50352, highly similar to Fetuin-B	2	1.0	1.3	1.0	1	
20.0	2	11.1	5.4	26072	B4DYU0	cDNA FLJ51403, highly similar to Sex hormone-binding globulin	6	1.0	1.3	0.6	1	
32.5	3	7.5	6.3	60405	B4E1D8	cDNA FLJ51597, highly similar to C4b-binding protein alpha chain	13	1.1	1.1	1.3	1	
204.7	22	32.7	5.8	78832	B7Z992	cDNA FLJ53698, highly similar to Gelsolin	82	0.8	1.0	1.0	1	
88.1	10	36.7	7.1	36494	B4E1I8	cDNA FLJ54228, highly similar to Leucine-rich alpha-2-glycoprotein	42	2.1	0.9	0.7	1	
7.1	1	0.9	8.2	95558	B4DSQ4	cDNA FLJ55365, highly similar to splicing factor, arginine/serine-rich14	3	1.0	0.8	0.9	1	
284.5	30	25.8	6.9	140943	B4E1Z4	cDNA FLJ55673, highly similar to Complement factor B 	178	1.1	1.0	0.9	1	
27.0	3	19	6.9	15718	B7Z8Q4	cDNA FLJ56652, highly similar to Hemopexin	3	1.1	1.1	1.0	1	
6.7	1	2.1	5.5	31673	B7Z8Q5	cDNA FLJ56762, highly similar to Hyaluronan-binding protein 2 	2	0.7	0.9	1.2	1	
14.9	2	4.5	6.2	47331	B4E1L6	cDNA FLJ56936, highly similar to Vitamin K-dependent protein S	5	0.7	1.0	1.0	1	
110.7	11	27	6.2	49757	B4E1H2	cDNA FLJ58564, highly similar to Plasma protease C1 inhibitor	105	1.3	1.0	0.9	1	
6.6	1	7	9.5	23961	B4DZ74	cDNA FLJ60198, highly similar to Homo sapiens RAD52 motif 1 	1	0.7	1.0	0.9	1	
7.6	1	0.9	6.4	95828	B4DK06	cDNA FLJ60610, highly similar to Dynamin-1 	1	0.3	0.5	0.3	1	
6.6	1	1.3	9.3	59105	B4DW29	cDNA FLJ61694, highly similar to Proteoglycan-4	1	1.3	2.0	2.8	1	
55.2	6	10.2	8.6	71354	A8K9A9	cDNA FLJ77744, highly similar to Homo sapiens kallikrein B, plasma 	10	0.9	0.8	1.1	1	
9.6	1	6.1	7.7	18332	B7ZAS5	cDNA, FLJ79289, highly similar to Extracellular matrix protein 1	1	1.0	0.8	1.0	1	
143.6	14	31.4	7.1	59512	B2R8I2	cDNA, FLJ93914, highly similar to Homo sapiens histidine-rich glycoprotein 	1	0.6	0.8	0.9	1	
323.6	31	41.3	5.5	115472	Q1L857	Ceruloplasmin	231	1.3	1.1	0.9	1	
15.2	2	3.3	6.5	65084	P06276	Cholinesterase 	3	0.6	0.9	1.1	1	
6.9	1	8.1	9.8	14494	Q7LAX7	CKII beta binding protein 2	1	2.7	1.0	1.8	1	
7.1	1	2.3	9.5	46586	O75122	CLIP-associating protein 2 (hOrbit2) 	1	1.2	1.3	1.0	1	
33.9	4	8.4	6.0	50063	P10909	Clusterin alpha chain 	8	1.2	1.1	1.0	1	
9.8	1	0.5	5.7	248655	P12259	Coagulation factor V light chain 	2	0.7	0.4	0.4	1	
61.0	7	12.5	8.1	67818	P00748	Coagulation factor XIIa light chain	17	1.0	1.3	1.1	1	
19.7	2	10.3	9.5	23688	P02745	Complement C1q subcomponent subunit A 	6	0.8	1.0	1.1	1	
27.1	3	13.3	9.0	23742	P02746	Complement C1q subcomponent subunit B 	6	0.9	0.8	1.0	1	
9.5	1	4.1	8.6	22813	P02747	Complement C1q subcomponent subunit C 	3	1.5	1.8	1.1	1	
45.4	6	8.3	5.8	78213	P00736	Complement C1r subcomponent light chain 	8	0.8	0.9	0.9	1	
22.5	3	6.6	6.3	49450	Q9NZP8	Complement C1r subcomponent-like protein 	2	1.2	0.8	1.2	1	
64.9	8	12.5	4.9	74887	P09871	Complement C1s subcomponent light chain 	15	0.9	0.8	1.0	1	
74.0	9	12.8	7.8	81085	P06681	Complement C2a fragment 	3	1.2	1.5	1.4	1	
275.0	31	20.5	6.1	188305	P01031	Complement C5 alpha' chain	96	1.3	1.0	0.9	1	
665.6	64	45.4	6.8	192802	B7ZVZ6	Complement component 4A 	12	1.1	0.9	0.9	1	
685.1	65	46.7	7.0	192749	B0UZ85	Complement component 4B 	4	1.3	1.0	1.3	1	
71.7	7	16.8	8.2	60140	B7Z550	Complement component 8	17	0.9	0.9	0.8	1	
685.4	65	46.7	7.0	192798	A2BHY4	Complement component C4B 	421	1.1	1.0	1.0	1	
137.5	14	20.5	6.2	102412	P13671	Complement component C6 	26	1.3	1.1	1.0	1	
114.2	12	19.9	6.2	91115	P10643	Complement component C7 	27	1.3	1.2	1.1	1	
53.3	5	12.3	5.8	61711	P07357	Complement component C8 alpha chain 	10	1.1	0.9	0.8	1	
68.8	7	56	8.9	20385	P07360	Complement component C8 gamma chain 	21	1.0	0.9	0.9	1	
111.1	12	25.1	5.5	60979	P02748	Complement component C9b 	44	1.6	1.1	0.8	1	
237.4	25	24.3	6.2	137053	P08603	Complement factor H 	87	1.0	1.0	1.1	1	
119.6	14	27.8	7.6	63457	P05156	Complement factor I light chain 	42	1.0	0.9	0.9	1	
6.6	1	2.1	5.8	42639	P08185	Corticosteroid-binding globulin 	2	1.0	1.3	1.0	1	
17.2	2	9.8	5.5	22950	P02741	C-reactive protein(1-205) 	3	41.1	4.4	5.1	1	
7.0	1	8.8	7.1	33681	P17544	Cyclic AMP-dependent transcription factor ATF-7	1	1.2	1.3	1.4	1	
6.4	1	1.2	5.8	112125	Q9BXX0	EMILIN-2 (Elastin microfibril interfacer 2) 	1	0.4	1.5	1.5	1	
6.0	1	5	9.1	50198	Q14222	Eukaryotic translation elongation factor 1 alpha 1	1	1.4	1.8	1.2	1	
180.0	19	11.1	5.6	239621	B7ZLF0	Fibronectin 1	41	0.4	0.9	1.4	1	
11.0	1	8.4	6.1	30228	Q15485	Ficolin-2 	1	0.7	0.8	0.6	1	
65.9	7	31.2	6.4	30354	O75636	Ficolin-3 	12	0.8	0.8	1.1	1	
204.7	21	57.4	6.5	49295	P02790	Hemopexin 	275	1.2	1.0	1.0	1	
77.7	9	16	6.4	54960	P05546	Heparin cofactor 2 	31	1.1	1.0	0.9	1	
150.8	15	35.5	7.1	57660	P04196	Histidine-rich glycoprotein 	97	0.6	0.8	0.8	1	
10.1	1	6.5	8.9	28980	A6XND1	Insulin-like growth factor binding protein 3 isoform b	1	0.6	2.1	1.1	1	
121.2	13	31.8	6.2	63247	P35858	Insulin-like growth factor-binding protein complex acid labile subunit (ALS) 	25	0.8	1.1	0.9	1	
266.4	26	36.5	6.6	103362	B2RMS9	Inter-alpha inhibitor H4	122	1.1	1.1	0.9	1	
155.2	15	29.8	6.4	71415	P19827	Inter-alpha-trypsin inhibitor heavy chain H1 	78	0.7	0.9	1.0	1	
259.9	28	45.1	5.8	72452	P19823	Inter-alpha-trypsin inhibitor heavy chain H2	157	0.7	1.0	0.9	1	
77.0	9	18.2	5.0	69360	Q06033	Inter-alpha-trypsin inhibitor heavy chain H3	14	1.6	1.2	0.6	1	
7.2	1	0.5	6.1	279048	Q7Z2Y8	Interferon-induced very large GTPase 1	1	1.0	0.5	1.1	1	
79.3	8	21.6	8.3	46355	P29622	Kallistatin 	18	0.6	1.0	0.9	1	
7.7	1	0.5	11.9	191307	O60382	KIAA0324	1	1.4	1.1	0.8	1	
204.0	21	50.1	6.6	47883	P01042	Low molecular weight growth-promoting factor	98	1.0	0.9	1.0	1	
105.8	11	42.8	6.3	36661	P51884	Lumican 	39	1.1	1.2	1.1	1	
7.0	1	2.3	9.3	58603	Q6B0I6	Lysine-specific demethylase 4D	1	1.2	1.2	1.0	1	
82.4	8	23.6	8.0	59980	Q96PD5	N-acetylmuramoyl-L-alanine amidase	25	0.7	1.0	0.8	1	
7.2	1	0.5	5.8	327549	Q5T321	Neurobeachin	1	0.5	1.4	0.4	1	
70.4	7	81.5	7.8	8865	P02775	Neutrophil-activating peptide 2(1-63)  	38	1.2	1.0	1.0	1	
6.8	1	1.6	6.5	47439	B6ZGT7	Nuclear receptor subfamily 2, group C isoform	1	1.0	1.3	1.3	1	
6.8	1	1.7	5.7	62837	Q96BF6	Nucleus accumbens-associated protein 2 	1	0.7	0.6	0.7	1	
9.1	1	3.7	5.6	29674	Q6UXB8	Peptidase inhibitor 16 	1	1.2	0.5	0.4	1	
141.6	15	42.9	6.1	44418	P36955	Pigment epithelium-derived factor 	43	1.0	0.9	1.0	1	
39.4	4	11.1	8.7	41424	P03952	Plasma kallikrein light chain 	2	0.9	1.5	0.7	1	
31.3	4	21.8	5.2	20575	P02753	Plasma retinol-binding protein(1-176) 	16	0.8	0.8	1.0	1	
205.0	20	30.2	7.3	88432	P00747	Plasmin light chain B 	86	1.0	1.1	1.0	1	
6.5	1	20	9.3	11061	Q9P150	PRO2760	1	1.0	1.0	1.1	1	
26.9	3	10.6	8.3	48462	Q9UK55	Protein Z-dependent protease inhibitor 	4	1.1	0.9	0.9	1	
7.2	1	4	5.8	19084	Q5T013	Putative hydroxypyruvate isomerase	7	1.6	3.6	3.0	1	
10.6	1	10.9	9.8	11333	C9JFR7	Putative uncharacterized protein CYCS	17	1.2	5.6	1.6	1	
39.4	4	21.6	6.7	29543	C9JPV4	Putative uncharacterized protein SERPINF2	1	0.9	1.0	1.1	1	
6.6	1	1.7	4.9	74071	P26374	Rab proteins geranylgeranyltransferase component A 2 	1	1.1	1.1	1.1	1	
68.0	8	36.3	6.5	23259	P02743	Serum amyloid P-component(1-203) 	24	1.0	0.8	0.9	1	
87.6	9	21.1	5.6	52278	P04004	Somatomedin-B 	72	0.9	1.0	1.1	1	
6.9	1	8.1	10.3	14203	B4E241	Splicing factor, arginine/serine-rich 3	1	0.8	1.0	0.9	1	
28.3	3	17.7	6.2	20169	P05452	Tetranectin 	9	0.7	0.9	0.8	1	
29.3	3	11.5	5.8	43944	A0PJG0	THBS1 protein	3	0.6	0.6	1.1	1	
189.3	18	42	5.3	65308	P00734	Thrombin heavy chain	91	0.9	1.1	1.0	1	
61.8	7	18	5.9	44102	P05543	Thyroxine-binding globulin 	14	0.7	1.1	1.1	1	
57.8	5	56.7	5.5	13761	P02766	Transthyretin 	30	0.7	0.9	0.7	1	
91.9	10	39.2	6.2	38999	P02760	Trypstatin	54	1.1	1.1	1.1	1	
6.7	1	0.5	5.9	255926	Q12923	Tyrosine-protein phosphatase non-receptor type 13	1	1.3	0.9	1.0	1	
7.0	1	1.6	7.6	50446	Q8N6V2	UBASH3A protein	2	1.1	1.2	1.1	1	
7.0	1	1.5	8.3	43395	Q15849	Urea transporter 2 	2	0.8	1.0	1.1	1	
11.0	1	2.5	7.3	69357	Q6EMK4	Vasorin 	1	0.7	0.7	1.0	1	
255.7	26	55	5.3	51243	P02774	Vitamin D-binding protein 	197	1.1	1.0	1.0	1	
167.2	17	56.5	5.7	32145	P25311	Zinc-alpha-2-glycoprotein 	101	1.2	1.0	1.0	1	
