# Supplementary material for: iTRAQ Identification of Candidate Serum Biomarkers Associated with Metastatic Progression of Human Prostate Cancer
Source: PLoS One. 2012 Feb 15;7(2):e30885. doi: 10.1371/journal.pone.0030885 (PMC3280251; doi:10.1371/journal.pone.0030885)
Supplement: Table S3 — Proteins differentially expressed between various cancer groups and BPH. (S3a) Proteins differentially expressed between the non-progressing and BPH group. (S3b), proteins differentially expressed between the progressing and BPH group. (S3c), proteins differentially expressed between the metastasis and BPH group. (RTF) [file pone.0030885.s005.rtf]

TABLE S3.  Proteins differentially expressed between various cancer groups and BPH.
Increased in non-progressing cancer relative to BPH						
Accession	Gene	Description	#unique peptides	% Cov	# peptides for quant	fold change	p-value 	
B7ZLF0	FN1	Fibronectin 1	19	11.1	41	1.4	3E-05	
P01023	A2M	Alpha-2-macroglobulin 	3	2.6	4	1.4	0.0042	
P43652	AFM	Afamin 	18	32.2	53	1.4	6E-09	
P02749	APOH	Beta-2-glycoprotein 1 	14	50.6	87	1.2	4E-07	
P51884	LUM	Lumican	11	42.8	39	1.1	0.0058	
P04004	VTN	Somatomedin-B 	9	21.1	72	1.1	0.0098	
P02765	AHSG	Alpha-2-HS-glycoprotein chain B	6	22.9	68	1.1	0.0079	
Decreased in non-progressing cancer relative to BPH						
P05546	SERPIND2	Heparin cofactor 2 	9	16	31	1.1	0.0092	
P19823	ITIH2	Inter-alpha-trypsin inhibitor heavy chain H2 	28	45.1	157	1.1	0.0002	
P01031	C5	Complement C5	31	20.5	96	1.1	0.0068	
P05156	CFI	Complement factor I light chain 	14	27.8	42	1.1	0.0031	
Q1L857	CP	Ceruloplasmin	31	41.3	231	1.1	0.0001	
B4E1H2	SERPING1	cDNA FLJ58564, similar to Plasma protease C1 inhibitor	11	27	105	1.1	0.0006	
P02743	APCS	Serum amyloid P-component	8	36.3	24	1.1	0.0016	
B4E1Z4	CFB	cDNA FLJ55673, highly similar to Complement factor B 	30	25.8	178	1.1	1E-06	
P02748	C9	Complement component C9b 	12	25.1	44	1.2	0.0037	
P01011	SERPINA3	Alpha-1-antichymotrypsin 	16	43.5	108	1.2	1E-04	
P04196	HRG	Histidine-rich glycoprotein 	15	35.5	97	1.3	4E-13	
P01008	SERPINC1	Antithrombin-III 	18	46.1	93	1.4	1E-11	
P02766	TTR	Transthyretin 	5	56.7	30	1.4	0.0002	
B4E1I8	LRG1	cDNA FLJ54228, similar to Leucine-rich alpha-2 glycoprotein	10	36.7	42	1.5	2E-05	
Q06033	ITIH3	Inter-alpha-trypsin inhibitor heavy chain H3 	9	18.2	14	1.7	0.0004	

S3a.  Proteins differentially expressed between the non-progressing and BPH group.


Increased in progressing cancer relative to BPH						
Accession	Gene	Description	#unique peptides	% Cov	# peptides for quant	fold change	p-value	
P01023	A2M	Alpha-2-macroglobulin (Alpha-2-M) 	3	2.6	4	1.9	0.0015983	
P00748	F12	Coagulation factor XIIa light chain	7	12.5	17	1.3	0.0002893	
P51884	LUM	Lumican (KSPG lumican) 	11	42.8	39	1.2	7.884E-05	
P10643	C7	Complement component C7 	12	19.9	27	1.2	0.0070035	
P02748	C9	Complement component C9b 	12	25.1	44	1.1	6.365E-05	
P04217	A1BG	Alpha-1B-glycoprotein 	16	41.8	129	1.1	3.729E-05	
P02749	APOH	Beta-2-glycoprotein 1 (Apo-H) 	14	50.6	87	1.1	0.0004938	
Q1L857	CP	Ceruloplasmin	31	41.3	231	1.1	2.91E-07	
P00747	PLG	Plasmin light chain B 	20	30.2	86	1.1	0.0003432	
B2RMS9	ITIH4	Inter-alpha (Globulin) inhibitor H4 	26	36.5	122	1.1	0.0026364	
P19823	ITIH2	Inter-alpha-trypsin inhibitor heavy chain H2 	28	45.1	157	1.0	0.0094065	
Decreased in progressing cancer relative to BPH						
P01042	KNG1	Kininogen 1	21	50.1	98	1.1	0.0001251	
P06727	APOA4	Apolipoprotein A-IV (Apo-AIV) (ApoA-IV) 	25	69.9	153	1.1	0.0001929	
P02765	AHSG	Alpha-2-HS-glycoprotein chain B 	5	18.8	15	1.1	0.0056273	
P00736	C1R	Complement C1r subcomponent light chain 	6	8.3	8	1.1	0.0095456	
B7ZLF0	FN1	Fibronectin 1	19	11.1	41	1.1	0.0094526	
P02743	APCS	Serum amyloid P-component(1-203) 	8	36.3	24	1.2	0.0002512	
P04196	HRG	Histidine-rich glycoprotein (HPRG) 	15	35.5	97	1.2	9.843E-13	
P02753	RBP4	Plasma retinol-binding protein(1-176) [CHAIN 3]	4	21.8	16	1.2	0.0008405	
S3b. Proteins differentially expressed between the progressing and BPH group.


S3c. Proteins differentially expressed between the metastasis and BPH group.
* = increased in all metastatic, progressing and non-progressing groups relative to BPH.
Increased in metastasis relative to BPH						
Accession	Gene	Description	#unique peptides	% Cov	# peptides for quant	fold change	p-value 	
B4E1I8	LRG1	cDNA FLJ54228, similar to Leucine-rich alpha-2 glycoprotein	10	36.7	42	2.1	2E-13	
P01011	SERPINA3	Alpha-1-antichymotrypsin 	16	43.5	108	1.8	4E-31	
Q06033	ITIH3	Inter-alpha-trypsin inhibitor heavy chain H3 	9	18.2	14	1.6	0.0002	
P02748	C9	Complement component C9b 	12	25.1	44	1.6	2E-08	
B4E1H2	SERPING1	cDNA FLJ58564, similar to Plasma protease C1 inhibitor	11	27	105	1.3	1E-10	
Q1L857	CP	Ceruloplasmin	31	41.3	231	1.3	1E-26	
P10643	C7	Complement component C7 	12	19.9	27	1.3	0.0016	
P01019	AGT	Angiotensin-3 	11	31.6	70	1.3	3E-05	
P13671	C6	Complement component C6 	14	20.5	26	1.3	9E-06	
P01031	C5	Complement C5	31	20.5	96	1.3	1E-08	
P25311	AZGP1	Zinc-alpha-2-glycoprotein 	17	56.5	101	1.2	4E-13	
P02790	HPX	Hemopexin	21	57.4	275	1.2	9E-15	
P02775	PPBP	Neutrophil-activating peptide 2	7	81.5	38	1.2	0.0003	
P06727	APOA4	Apolipoprotein A-IV 	25	69.9	153	1.2	6E-14	
P02749*	APOH	Beta-2-glycoprotein 1 	14	50.6	87	1.2	7E-05	
B2RMS9	ITIH4	Inter-alpha (Globulin) inhibitor H4 	26	36.5	122	1.1	1E-07	
P04217	A1BG	Alpha-1B-glycoprotein 	16	41.8	129	1.1	0.0008	
A2BHY4	C4B	Complement component C4B 	65	46.7	421	1.1	5E-10	
B4E1Z4	CFB	cDNA FLJ55673, similar to Complement factor B	30	25.8	178	1.1	0.0003	
Decreased in metastasis relative to BPH						
P00734	F2	Thrombin heavy chain 	18	42	91	1.1	0.0026	
P02753	RBP4	Plasma retinol-binding protein	4	21.8	16	1.2	0.0002	
P00736	C1R	Complement C1r subcomponent light chain 	6	8.3	8	1.2	0.0065	
P35858	IGFALS	Insulin-like growth factor-binding protein complex	13	31.8	25	1.3	0.0012	
B7Z992	GSN	cDNA FLJ53698, highly similar to Gelsolin	22	32.7	82	1.3	1E-13	
P19827	ITIH1	Inter-alpha-trypsin inhibitor heavy chain H1 	15	29.8	78	1.3	4E-09	
P19823	ITIH2	Inter-alpha-trypsin inhibitor heavy chain H2  	28	45.1	157	1.4	3E-26	
Q96PD5	PGLYRP2	N-acetylmuramoyl-L-alanine amidase 	8	23.6	25	1.4	5E-06	
P05543	SERPINA7	Thyroxine-binding globulin 	7	18	14	1.4	0.0012	
P02766	TTR	Transthyretin 	5	56.7	30	1.5	5E-05	
P05452	CLEC3B	Tetranectin 	3	17.7	9	1.5	0.0002	
P04196	HRG	Histidine-rich glycoprotein	15	35.5	97	1.6	9E-31	
P29622	SERPINA4	Kallistatin (PI-4) 	8	21.6	18	1.6	0.0002	
P43652	AFM	Afamin 	18	32.2	53	2.1	5E-22	
P02765	AHSG	Alpha-2-HS-glycoprotein 	6	22.9	68	2.1	2E-21	
B7ZLF0	FN1	Fibronectin 1	19	11.1	41	2.5	5E-13	
