# Supplementary material for: iTRAQ Identification of Candidate Serum Biomarkers Associated with Metastatic Progression of Human Prostate Cancer
Source: PLoS One. 2012 Feb 15;7(2):e30885. doi: 10.1371/journal.pone.0030885 (PMC3280251; doi:10.1371/journal.pone.0030885)
Supplement: Table S4 — Proteins differentially expressed between the progressing versus non-progressing group. (RTF) [file pone.0030885.s006.rtf]

TABLE S4. Proteins differentially expressed between the progressing versus non-progressing group.

Increased in progressing relative to non-progressing cancer						
Accession	Gene	Description	#unique peptides	% Cov	# peptides for quant	fold change	p-value 	
Q06033	ITIH3	Inter-alpha-trypsin inhibitor heavy chain H3 	9	18.2	14	2.0	0.00043	
B4DYU0	SHBG	cDNA FLJ51403, highly similar to Sex hormone-binding globulin	2	11.1	6	1.9	0.0056	
B4E1I8	LRG1	cDNA FLJ54228, similar to Leucine-rich alpha-2-glycoprotein	10	36.7	42	1.4	1.1E-05	
P02748	C9	Complement component C9b	12	25.1	44	1.4	5.1E-07	
P01008	SERPINC1	Antithrombin-III 	18	46.1	93	1.2	1E-05	
Q1L857	CP	Ceruloplasmin	31	41.3	231	1.2	4.1E-15	
P01011	SERPINA3	Alpha-1-antichymotrypsin His-Pro-less 	16	43.5	108	1.2	9E-05	
B2RMS9	ITIH4	Inter-alpha (Globulin) inhibitor H4 	26	36.5	122	1.1	4.6E-05	
B4E1H2	SERPING1	cDNA FLJ58564, highly similar to Plasma protease C1 inhibitor	11	27	105	1.1	0.00023	
P04217	A1BG	Alpha-1B-glycoprotein	16	41.8	129	1.1	0.00084	
P10643	C7	Complement component C7 	12	19.9	27	1.1	0.0011	
B4E1Z4	CFB	cDNA FLJ55673, highly similar to Complement factor B 	30	25.8	178	1.1	2.9E-05	
P01031	C5	Complement C5 	31	20.5	96	1.1	0.00828	
Decreased in progressing relative to non-progressing cancer						
P01042	KNG1	Kininogen 1	21	50.1	98	1.1	1.9E-05	
P02749	APOH	Beta-2-glycoprotein 1 	14	50.6	87	1.1	0.00597	
P06727	APOA4	Apolipoprotein A-IV 	25	69.9	153	1.1	1.7E-08	
P02765	AHSG	Alpha-2-HS-glycoprotein chain B	6	22.9	68	1.2	1E-05	
P04004	VTN	Somatomedin-B 	9	21.1	72	1.2	0.00073	
P02753	RBP4	Plasma retinol-binding protein	4	21.8	16	1.2	1.9E-06	
B0UZ85	C4B	Complement component 4B 	65	46.7	4	1.3	0.00837	
O75636	FCN3	Ficolin-3 	7	31.2	12	1.4	0.00246	
A8K9A9	KLKB1 	cDNA FLJ77744, highly similar to  kallikrein B	6	10.2	10	1.4	0.00109	
Q9NZP8	C1RL	Complement C1r subcomponent-like protein	3	6.6	2	1.4	0.00776	
P43652	AFM	Afamin 	18	32.2	53	1.5	2.5E-10	
B7ZLF0	FN1	Fibronectin 1	19	11.1	41	1.6	2E-09	
